# Safety and Effectiveness of Conversion from Adjustable Gastric Band to Ring Augmented Roux-en-Y Gastric Bypass

**DOI:** 10.1007/s11695-025-08463-7

**Published:** 2026-01-14

**Authors:** Kayleigh Ann Martina van Dam, Geert Henricus Jozef Martinus Verkoulen, Evelien de Witte, Pieter Petrus Henricus Luciën Broos, Jan Willem M. Greve, Evert-Jan Gijsbert Boerma

**Affiliations:** 1 Bariatric Surgery, Zuyderland Medisch Centrum, Heerlen, Netherlands; 2 NUTRIM, Maastricht University, Maastricht, Netherlands; 3 Nederlandse Obesitas Kliniek, Heerlen, Netherlands; 4 NUTRIM, Maastricht University, Maastricht, Netherlands

**Keywords:** Adjustable gastric band, Ring augmented Roux-en-Y gastric bypass, MiniMizer, Conversional surgery

## Abstract

**Background:**

Laparoscopic Adjustable Gastric Band (AGB) has shown suboptimal long-term results with a non-success rate of 20–56% with an accompanying removal rate of 10–50% due to suboptimal clinical response or complications. Conversion to RYGB is proven to be a safe and effective option. However, current literature contains no studies which use additional placement of a silicone ring (MiniMizer) around the pouch. Therefore, this study aims to evaluate the safety and effectiveness of conversion from AGB to ring augmented RYGB (raRYGB).

**Methods:**

All consecutive laparoscopic AGB to raRYGB conversions performed between January 2016 and October 2023 were included. All procedures were performed by a one-stage approach. The primary outcome was percentage total weight loss (%TWL) after 1-year follow-up. Secondary outcomes consisted of %TWL after 2, 3, 4, and 5 years, cumulative %TWL, and early and late complications.

**Results:**

We included a total of 240 patients of whom 195 were female (81.3%). Mean pre-conversion BMI was 40.3 kg/m^2^. The average %TWL 1- and 5-year after the conversion was 25.4% and 18.9%. Cumulative %TWL, calculated from before AGB, was 33.7% after 1 and 30.2% after 5 years. 8 complications occurred within 30 days, 3 of which were *≤* CD3a and 5 *≥* CD3b. A total of 8 MiniMizers were removed.

**Conclusion:**

Conversion from laparoscopic AGB to raRYGB is a valid one-stage conversion method with significant weight loss after 1 and durable weight loss up to 5 years of follow-up. The short- and long-term complication rate is acceptable and ring-specific complications are rare.

## Key Points

• Single-stage conversion from AGB to ring-augmented RYGB is a safe and effective conversional option.

• This approach achieves substantial weight loss at 1 years and maintains durability up to 5 years.

• Ring-related adverse events were rare, underscoring the safety of the MiniMizer.

## Introduction

The global prevalence obesity continues to rise causing major health issues [1, 2]. Metabolic Bariatric Surgery (MBS) remains the most effective treatment for sustained weight loss and resolution of obesity-related comorbidities [3]. Nowadays, the most common procedures are Roux-en-Y Gastric Bypass (RYGB) or Sleeve Gastrectomy (SG). Adjustable Gastric Banding (AGB) was frequently performed, however due to suboptimal long-term results AGB has become mostly outdated [4]. Long-term studies report non-success rates of 20–56% and removal rates of 10–50% due to suboptimal clinical response or complications [4–6]. Common causes of non-success are pouch dilatation or band slippage [5].

In patients requiring surgery, conversion to RYGB, SG, or biliopancreatic diversion with duodenal switch is a common approach [5, 7]. Conversion to RYGB is widely accepted as preferred procedure and has been proven safe and effective [4, 5, 7]. However, studies generally evaluate conversion to standard RYGB without (adjustable) band or ring. Lecot et al. showed that retaining the adjustable AGB led to frequent reoperations, concluding removal is necessary [4]. To date, no studies investigating the conversion from AGB to RYGB have evaluated the use of a non-adjustable silicone ring (e.g. MiniMizer).

Ring-augmented RYGB (raRYGB), in which a silicone ring is placed around the pouch, has shown greater weight loss and reduced recurrent weight gain than standard RYGB in primary surgery [8–10]. Additionally, raRYGB is proven safe and effective in secondary settings, such as conversion after SG [11]. Cumulative %TWL, calculated from the initial procedure, after these conversions is comparable to %TWL achieved after a primary raRYGB procedure [11, 12].

Therefore, the aim of the present study was to evaluate the safety and effectiveness of conversion from AGB to raRYGB regarding weight loss and complications.

## Methods

### Patient Selection

All consecutive patients who underwent conversion from AGB to raRYGB between January 2016 and October 2023 at Hospital X were retrospectively included. Conversion indications included recurrent weight gain, band-related problems (e.g. slippage or pouch dilatation), and functional problems such as GERD. Patients were categorized into groups: (1) recurrent weight gain, (2) band/functional problems, and (3) combined indications. All were assessed by a multidisciplinary team. Approval was granted by the local ethics committee in accordance with the 2013 Declaration of Helsinki.

### Surgical Procedure

All procedures were performed laparoscopically using five trocars. The gastric band and fibrous capsule were completely removed. An 8–10 cm long pouch was created over a 40 French orogastric tube. Hiatal hernias, if present, were repaired by cruroplasty. The jejenum was identified at the ligament of Treitz and a 60 cm biliopancreatic limb was measured. This limb was brought antecolically and antegastrically to the gastric pouch and a linear stapled 30–40 mm end-to-side gastrojejunal anastomosis was created. The stapling defect was closed using Vloc (Medtronic, USA). The biliopancreatic limb was transected and a linear stapled 40–60 mm side-to-side jejunojejunal anastomosis was created with an alimentary limb of 120 cm. Limb lengths followed standard approach used for primary and revisional RYGB procedures. Mesenteric defects at the entero-enterostomy and Petersen site were routinely closed using endoclips (EHMS from Johnson&Johnson or EndoHernia Stapler Universal from Medtronic) A silicone ring, the MiniMizer (Bariatric Solutions, Switzerland), was placed around the pouch. The MiniMizer was placed at least 2 cm above the gastrojejunal anastomosis and at least 2 cm below the gastroesophageal junction. The closing position was between 7 and 8 cm, typically at 7.5 cm. The MiniMizer was fixated on the vertical staple line of the pouch with a non-absorbable suture (Prolene, Ethicon, USA).

### Data Collection

Data was collected from electronic patient files. Baseline data included age, gender, height, weight, BMI, time between procedures and indication for conversion. In addition, weight and BMI at initial AGB screening were collected. The primary outcome was %TWL after 1-year follow-up, calculated from pre-conversion weight. Secondary outcomes included %TWL at 2–5 years, cumulative %TWL, and early (< 30 days) and late (> 30 days) complications. Complications included general and ring related complications.

Cumulative %TWL was calculated using initial weight during screening for AGB. Complications were classified using the Clavien-Dindo system [13].

### Statistical Analysis

Statistical analysis was performed using IBM SPSS Statistics v29. Categorical variables were presented as frequencies with percentages. Continuous variables were presented as mean *±* standard deviation (SD) in case of normal distributed variables and as median and inter-quartile-range (IQR) in case of skewed distributed variables. Differences between subgroups were tested using One-way ANOVA for three groups, followed by post-hoc analysis to identify pairwise differences. A *p*-value of < 0.05 was considered statistically significant.

## Results

A total of 240 patients were analyzed, of whom 195 were female (81.3%). Baseline characteristics are summarized in Table 1. The group had a mean age of 49 *±* 9.7 years and mean preoperative BMI was 40.3 kg/m^2^ (*±* 5.8).

**Table 1:** Baseline characteristics

Variables	*N* = 240
Age (years)	49 *±* 9.7
Gender	
* Male*	45 (18.8)
* Female*	195 (81.3)
Height (cm)	168.6 *±* 0.9
Weight at initial procedure (kg)	128.7 *±* 19.8
BMI at initial procedure (kg/m^2^)	45 *±* 5.5
Weight at screening for conversion (kg)	114.2 *±* 19.5
BMI at screening for conversion (kg/m^2^)	40.3 *±* 5.8

Data are presented as mean ± standard deviation or N (%)

Primary indications for conversion were recurrent weight gain (76.6%), band/functional problems (11.7%) and a combination of both (11.7%). On average, 11 years (8–15) had elapsed between initial AGB and conversion (Table 2). Conversion to RYGB had a median operating time of 93 min (77–115). In terms of surgical technique, 86.7% of patients received a MiniMizer with a closing position of 7.5 cm, while the remainder received a MiniMizer with 7–8 cm closing position.

**Table 2:** Surgery details

Variables	*N* = 240
Time between AGB and conversion (years)	11 (8–15)
Indication for conversion	
* Recurrent weight gain*	184 (76.6)
* Band/functional problems*	28 (11.7)
* Band/functional problems and recurrent weight gain*	28 (11.7)
Surgery duration (min)	93 (77–115)
Closing position MiniMizer	
7 cm	23 (9.6)
7.5 cm	208 (86.7)
8 cm	4 (1.7)

Data are presented as median (IQR) or N (%)

### Weight Loss Outcomes

Mean BMI at AGB screening was 45 kg/m^2^ (*±* 5.5) while mean BMI at screening for conversion was 40.3 kg/m^2^ (*±* 5.8). One-year follow-up was reached by all patients while data was available for 228/240 (95%) of patients. Not all time points were reached by the patients and follow-up data was available in 87.4%, 58.2%, 65.7% and 55% after 2-, 3-, 4-, and 5-years respectively (Table 3).

**Table 3:** %TWL and cumulative %TWL during 5-year follow-up after conversion

	Follow-up	%TWL	cumulative %TWL (from primary procedure)
Pre-conversion			10.6 *±* 11.6
3-month follow-up	237/240 (98.8)	15.3 *±* 6.1	24.4 *±* 9.8
6-month follow-up	234/240 (97.5)	21.2 *±* 7.8	30 *±* 9.4
1-year follow-up	228/240 (95)	25.4 *±* 10.2	33.7 *±* 10.3
2-year follow-up	188/215 (87.4)	25.3 *±* 11.7	33.8 *±* 11.3
3-year follow-up	124/213 (58.2)	23.3 *±* 11.4	31.6 *±* 10.8
4-year follow-up	90/137 (65.7)	21.2 *±* 12.1	30.6 *±* 10.1
5-year follow-up	55/100 (55)	18.9 *±* 10.6	30.2 *±* 10.4

Follow-up based on number of patients who reached the specific time-point. %TWL is calculated based on weight at screening for conversion (*n* = 240), cumulative %TWL is calculated based on weight for initial AGB (*n* = 201)

Of the 240 patients, 212 underwent conversion due to recurrent weight gain, with or without band-related issues. Among these patients, the lowest weight achieved after AGB was available for 146 patients. In this subgroup, %TWL from AGB to lowest recorded weight was 30.1% (21.2–37.5). Prior to conversion patients had regained a substantial portion of their lost weight, with a median regain of 25.5% (15-40.1).

Following conversion, one-year %TWL, calculated for all indications, was 25.4%*±*10.2 (Table 3). During follow-up %TWL was 25.3 *±* 11.7, 23.3 *±* 11.4, 21.2 *±* 12.1, and 18.9 *±* 10.6 at respectively 2-, 3-, 4-, and 5-year follow-up. In addition, Table 3 presents cumulative %TWL using the start weight from the initial AGB procedure. At the moment of screening for conversion, patients had a mean %TWL of 10.6% (*±* 11.6). After 1-year follow-up t cumulative %TWL was 33.7 *±* 10.3. and after 5 years 30.2 *±* 10.4. The maximum follow-up duration considered was 5 years, according to the Dutch Obesity Clinic protocol. The mean maximum follow-up time was 4.6 years with a cumulative %TWL of 32.3 *±* 10.9.

**Fig. 1:**
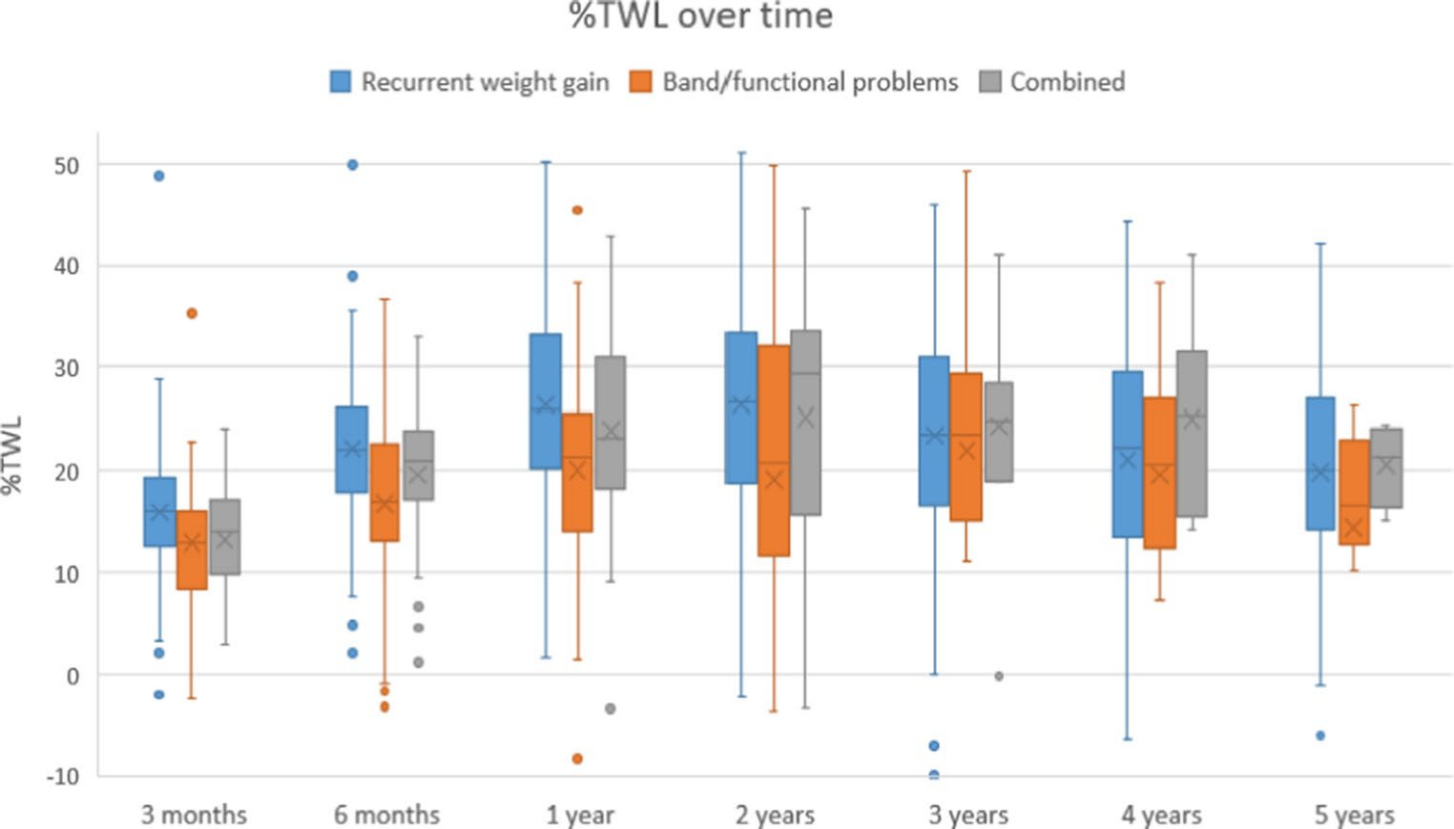
%TWL over 5-year follow-up

**Fig. 2:**
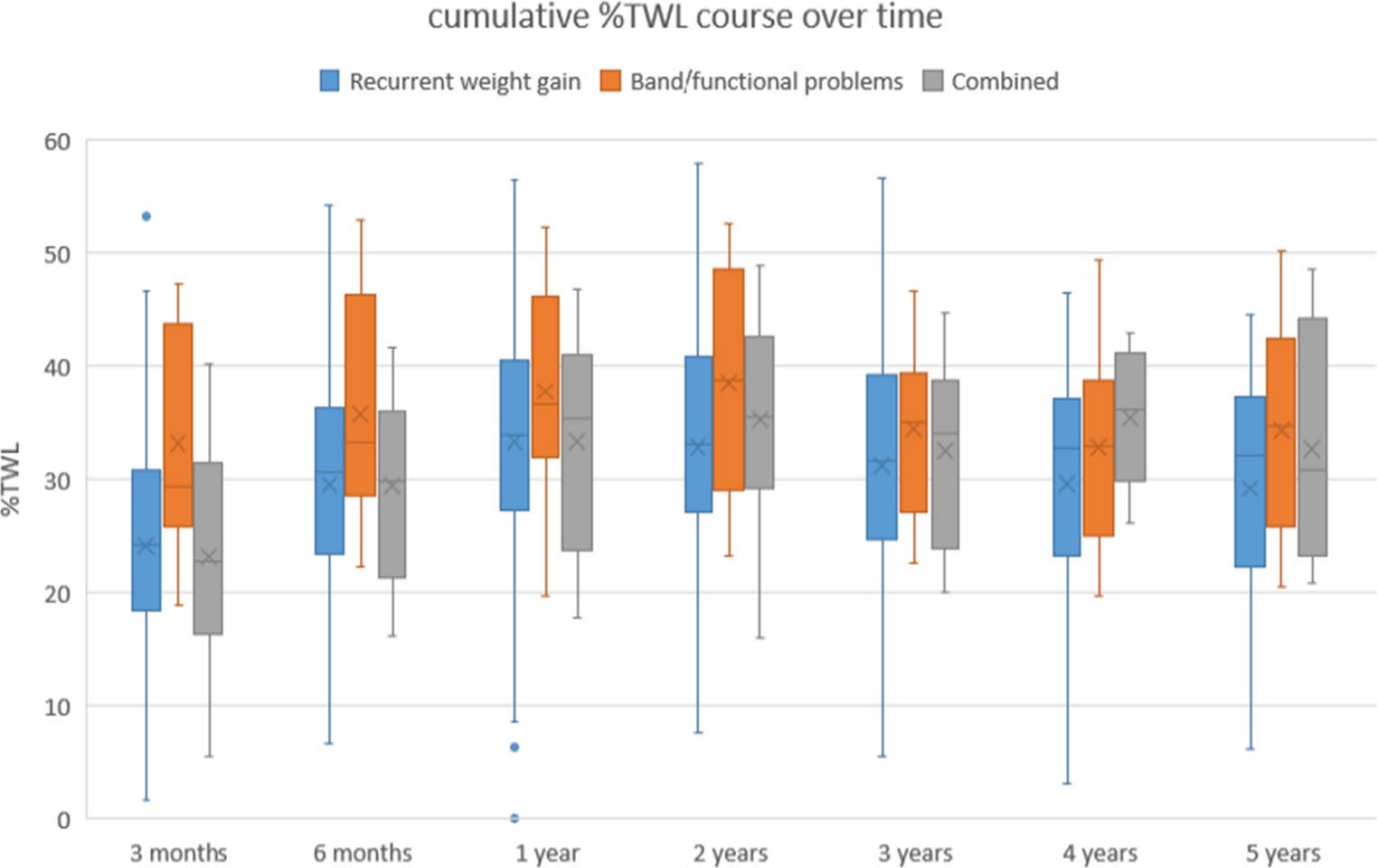
cumulative %TWL over 5-year follow-up

Weight loss outcomes were also compared across the indication subgroups (Figs. 1 and 2). Up to 2-year follow-up, a statistically significant difference in %TWL was observed between the groups (ANOVA, *p* < 0.05). Post-hoc analysis revealed that patients converted for recurrent weight gain achieved significantly greater %TWL compared to those converted for band/functional problems at 1 year (26.3 *±* 9.3 vs. 20.1 *±* 13.7, *p* = 0.010). No statistically significant differences were found with the combined and recurrent weight gain group at any timepoint. From two years and onward no statistical differences in %TWL were found between any of the groups (*all post-hoc p > 0.05*). At 5-year follow-up, the recurrent weight gain group had a mean %TWL of 19.9 *±* 10.3, the band/functional group of 14.5 *±* 12.9 and the combined group of 20.6 *±* 4.1 (*p* = 0.325).

## Complications

A total of 41 short- and long-term complications were observed in 40 patients (16.7%) (Tables 4 and 5). Of these complications, eight (3.3%) occurred within the first 30 days postoperatively. According to the Clavien-Dindo classification, these included three classified as < CD3a and five as *≥* CD3b. Short-term complications consisted of anastomotic leakages (*n* = 4), all treated laparoscopically. There was one case of major bleeding which required laparotomy. Additionally, a gastro-gastric fistula and a case of food impaction were treated endoscopically, while one minor bleeding was treated conservatively with tranexamic acid.

**Table 4:** Short- and long-term complications

Variables	*N* = 240
MiniMizer in situ at last FU	232 (96.7)
MiniMizer related complications	
* Ring slippage*	0
* Ring erosion *	1 (0.4)
* Dysphagia *	1 (0.4)
Patients with complications	40 (16.7)
Short-term complications	
Patients with short-term complications	8 (3.3)
Short-term (< 30 days) complications according to	
Clavien-Dindo	
* 2*	1 (0.4)
* 3a*	2 (0.8)
* 3b*	5 (2.1)
Long-term complications	
Patients with long-term complications	33 (13.8)
Long-term (>30 days) complications according to	
Clavien-Dindo	
* 2*	2 (0.8)
* 3a *	4 (1.7)
* 3b*	26 (10.8)

**Table 5:** Types of complications

	*N* = 240
Short-term	
Anastomotic leakage	4 (1.7)
Bleeding	2 (0.8
Gastro-gastric fistula	1 (0.4)
Food impaction	1 (0.4)
Long-term	
Internal herniation	13 (5.4)
Pouch-related problems	5 (2.1)
Marginal ulcers	5 (2.1)
Diagnostic laparoscopies	3 (1.3)
Impending blow-out of excluded stomach	1 (0.4)
Food impaction	1 (0.4)
Gastro-gastric fistula	1 (0.4)
Hematoma	1 (0.4)
Stenosis	1 (0.4)
*MiniMizer related *	
Dysphagia	1 (0.4)
Erosion	1 (0.4)

Long-term complications, occurring after more than 30 days, were observed in 32 patients. The most frequent complication was internal herniation (*n* = 13) which were all treated laparoscopically. The majority of internal herniations occurred at the entero-enterostomy site. A few occurred at Petersen defect and one patient developed an internal herniation due to adhesions despite proper closure of both mesenteric defects. Other complications included diagnostic laparoscopies for unexplained abdominal pain (*n* = 3), pouch-related revisions with repositioning of the MiniMizer (*n* = 5) and stenosis of gastrojejunostomy requiring revision surgery (*n* = 1). Marginal ulcers were observed in 5 patients, some of whom treated conservatively with proton pump inhibitors (*n* = 2) and some who presented with perforations and were managed laparoscopically (*n* = 2). In addition, one patient with a marginal ulcer required surgical revision of the gastro-enterostomy. Additional long-term complications included food impaction (*n* = 1) treated gastroscopically, and hematoma (*n* = 1) and a gastro-gastric fistula (*n* = 1), both managed laparoscopically. The MiniMizer was removed in two cases, due to erosion and dysphagia. Additionally, adhesiolysis was performed during diagnostics laparoscopy in one patient, and another’s entero-enterostomy was revised due to an impending blow-out of the excluded stomach.

The Minimizer was removed in eight patients (3.3%). Two (0.8%) experienced MiniMizer-related complications. One had dysphagia without visible abnormalities. The other was due to erosion of the MiniMizer which was treated by endoscopic removal. In addition, six MiniMizers (2.5%) were removed as part of surgical treatment for other complications. These complications included anastomotic leakage (*n* = 2), perforation of the gastrojejunostomy due to marginal ulcer (*n* = 2), gastro-gastric fistula (*n* = 1), and stenosis of the gastrojejunostomy (*n* = 1).

## Discussion

This study evaluated the outcomes of a large series of conversion from AGB to raRYGB. Mean %TWL at 1-year follow-up was 25.4%, with a cumulative %TWL of 33.7%. Cumulative %TWL after 1-year is comparable to the 35.6% TWL after primary raRYGB as shown by Jense et al. [8]. Weight loss results remain stable during five years and are comparable with the 30–35% TWL after primary raRYGB [8]. Additionally, cumulative outcomes after conversion to raRYGB are slightly higher than most reported outcomes after standard RYGB [8, 14, 15]. Compared to standard RYGB weight loss outcomes reported in literature of 30% after 1 year and 27.7% after 5 years, cumulative TWL of 33.7% and 30.2% demonstrates comparable weight loss results following conversion to raRYGB [8, 14]. In addition, these results are similar to those reported by Okkema et al. although their study did not demonstrate a clear advantage of raRYGB over standard RYGB [15].

When comparing current results to studies evaluating conversion from AGB to RYGB, standard 25.4% TWL is comparable to the range of 24.8–33.5% reported in literature [4, 16, 17]. However, these studies calculated %TWL using initial weight prior to AGB. This corresponds to the cumulative %TWL in the present study. When comparing cumulative outcomes, 33.7% TWL is higher than those reported by Pujol-Rafols et al. (30.4%) and Creange et al. (24.8%) [16, 17]. The study by Lecot et al. reported a comparable cumulative %TWL of 33.5%, although in this cohort the adjustable band was left around the pouch [4].

Subgroup analysis based on conversion indication revealed significant differences in weight loss up to two years. Patients who underwent conversion due to recurrent weight gain achieved significantly greater %TWL in the first postoperative year compared to the band/functional indication group (26.3 vs. 20.1). However, after two years this difference was no longer significant. Although patients in the combined group might be expected to have less %TWL due to the presence of multiple indications, there were no significant differences between recurrent weight gain and the combined group. The relatively small sample size of the combined group (11.7%) compared to the recurrent weight gain group (76.6%) may have limited the ability to detect minor differences between groups. Observed early differences in %TWL between subgroups may reflect differences in patient motivation or severity of initial recurrent weight gain. The convergence of weight loss outcomes after two years suggests that, regardless of initial indication, conversion to raRYGB provides comparable long-term effectiveness.

This study showed that short-term complications occurred in 3.3% of the patients. Long-term complications occurred in 13.8% of the patients. MiniMizer related complications were rare as only two patients (0.8%) required removal due to ring-specific issues. An additional six rings were removed during surgical management of unrelated complications such as leakage or fistula. These findings highlight that ring removal is rarely due to device failure or intolerance. The literature reports more substantial long-term risks associated with ring placement. The 5-year randomized controlled trial by Okkema et al. reported a ring removal rate of 12%, primarily due to dysphagia [15]. However, this study focused on primary procedures and may have used a tighter ring circumference than applied in our revisional cohort. In contrast, a recent study conducted in our center reported 4.4% ring-related complications and a 2.4% removal rate, which aligns more closely with our findings [18].

In the current study, overall short-term complication rate is lower than the rates reported specific for conversion from AGB to RYGB. Literature shows a complication rate between 4 and 10% for either one- or two-stage AGB to RYGB conversion [4, 19, 20]. This rate is comparable to the complication rate of 2.6% following secondary procedures reported by the Dutch Audit for Treatment of Obesity (DATO) [21].

In addition to the short-term complication rate, this study provides insights into long-term safety outcomes. A total of 13.8% had a complication after the 30-day period. Regarding severe complications (CD *≥* 3b) long-term complication rate was 10.8%. After primary RYGB with long-term follow-up of more than ten years, a range from 19.5% to 23% complication rate was found [22, 23]. The weighted late complication rate in the meta-analysis by Buchwald et al. was 20% [24]. Among these studies the majority was due to internal herniation, as is the case in our study. A three-year follow-up after either primary RYGB or SG showed a slightly lower complication rate of 11% [25]. Our rate of 10.8% is comparable to these primary MBS long-term results. The rate is also comparable to published long-term complication rate after conversional MBS as a 15% long-term complication rate was found after conversion from AGB to SG or RYGB [5].

A notable feature of our surgical strategy was the single-stage approach, where band removal and conversion to raRYGB are performed during the same procedure. Some centers prefer a two-stage approach to reduce perioperative risks, especially in cases with severe adhesions. Our findings demonstrate that a single-stage approach can be performed safely. The early complication rate of 3.3% is lower than those reported in previous studies [4, 19, 20]. In recent literature, there is more evidence that shows no difference in complications between one- or two-stage conversions [15, 26, 27].

### Limitations

This retrospective study has several limitations. First, although 1-year follow-up rates were high with 95%, the follow-up decreased over time to 50% after 5 years. This loss to follow-up is consistent with trends observed in MBS literature where long-term follow-up has drastically decreased to 10%-29% [28–31]. However, reduced follow-up may introduce bias, particularly in assessing weight loss outcomes. Second, the use of a ring is standard practice in primary and secondary RYGB procedures at our center. As a result, a control group was not available for comparison. This absence limits the ability to directly attribute outcomes to ring augmentation and should be considered when interpreting the results. Third, subgroup analysis was performed using univariate methods. Multivariate adjustment was not applied due to the retrospective design and limited sample sizes in some subgroups, which may reduce statistical power. As a result, subgroup differences should be considered exploratory.

## Conclusion

This study demonstrates that conversion from AGB to raRYGB using a single-stage approach is safe and effective. Cumulative weight loss outcomes are comparable to those of primary raRYGB and standard RYGB. The short-term complication rate of 3.3% and long-term rate of 13.8% are acceptable compared to the literature. Ring-specific adverse events were rare (0.8%), supporting the MiniMizer’s safety in conversional surgery.

## Data Availability

No datasets were generated or analysed during the current study.
